# Pigment epithelium-derived factor (PEDF) represses the glucose transporter 1 (*GLUT1*) mRNA expression and may be a potential therapeutic agent in psoriasis: a case–control and experimental study

**DOI:** 10.1038/s41598-023-48565-9

**Published:** 2023-12-05

**Authors:** Khalid M. Mohany, Sherouk Elkady, Eman M. Kamal Youssef, Noorhan M. Sayed, Naglaa Kamal Idriss

**Affiliations:** 1 Medical Biochemistry and Molecular Biology Department, Faculty of Medicine, Assiut University, Assiut, 71515 Egypt; 2 Department of Dermatology, Venereology, and Andrology, Assiut University Hospital, Faculty of Medicine, Assiut University, Assiut, Egypt; 3 Faculty of Medicine, Assiut University, Assiut, Egypt

**Keywords:** Biochemistry, Biological techniques, Cell biology, Chemical biology, Molecular biology, Biomarkers, Diseases, Medical research, Molecular medicine

## Abstract

We investigated the whole blood *GLUT1* mRNA expression and serum pigment epithelium-derived factor (PEDF), interleukin-6 (IL-6), fetuin-A, and pentraxin-3 (PTX3) levels in psoriatic patients and tested their correlations with the severity of psoriasis using the psoriasis area and severity index (PASI) score. Also, we tested the *GLUT1* mRNA expression after an in vitro treatment of human skin fibroblast (HSF) cell lines with PEDF. The case–control part of the study recruited 74 participants (44 psoriatic patients and 30 healthy volunteers). Whole blood *GLUT1* mRNA fold changes were estimated by RT-PCR, and serum PEDF, IL-6, fetuin-A, and PTX3 levels were measured by ELISA kits. In the experimental part, the HSF cell lines were treated with different concentrations of PEDF for different times to test its effect on the *GLUT1* mRNA expression. The whole blood *GLUT 1* expression significantly increased in psoriatic patients and correlated positively with serum IL-6, fetuin-A, PTX3 levels and with the severity of psoriasis while negatively with serum PEDF levels. The PEDF-treated HSF cell lines showed a time- and dose-dependent decline in the *GLUT 1* mRNA expression. The whole blood *GLUT 1* mRNA is a non-invasive biomarker that is associated with the severity of psoriasis. PEDF represses *GLUT 1* expression and may be a potential therapeutic agent in psoriasis.

Trial registration: ClinicalTrials.gov Identifier: NCT04242082.

## Introduction

Psoriasis is a chronic non-communicable disfiguring skin disorder. It manifests as scaly plaques, pain, itching, and sometimes bleeding^1^. The prevalence of psoriasis is 2–4% worldwide^2^ while ranges from 0.19 to 3% in Egypt^3^.

The exact cause of psoriasis is still unknown, but immune-metabolic, inflammatory, and genetic factors are thought to play a role^4^. Glucose transporter-1 gene (*Glut1*) overexpression is an early step in the pathogenesis of psoriasis. Glut1 enhances epidermal hyperproliferation, inflammation, and angiogenesis and is associated with the psoriasis severity and its metabolic comorbidities^5,6^. Previous studies relied mainly on skin lesion biopsies to report data about GLUT1 and its gene expression^5–8^.

Pigment epithelium-derived factor (PEDF; a 50-kDa glycoprotein) is a member of the serine protease inhibitor gene family. It is encoded by Serpin Family F Member 1 (SERPINF1/EPC1) on chromosome 17p13.1^9^. It has an antiangiogenic effect. Topical application of PEDF on a mouse model of psoriatic disease reduced the skin proliferation and angiogenesis^10^.

Being immune-metabolic mediated, psoriatic patients were found to have higher levels of interleukin-6 (IL-6), fetuin-A, and pentraxin-3 (PTX3) than healthy individuals^11–15^.

The current work aimed to investigate the whole blood *GLUT1* mRNA expression and serum PEDF, IL-6, fetuin-A, and PTX3 levels in psoriatic patients and tested their correlations with the severity of psoriasis using the psoriasis area and severity index (PASI) score. Also, the study tested the *GLUT1* mRNA expression after an in vitro treatment of HSF cell lines with different concentrations of PEDF for different times.

## Materials and methods

The current study was conducted in the Medical Biochemistry Department, Faculty of Medicine, Assiut University, in collaboration with Dermatology Department, Assiut University Hospital. The study was reviewed and authorized by the local ethics committee, Faculty of Medicine, Assiut University (IRB: 17101042). It is carried out in compliance with the Declaration of Helsinki^16^.

### Sample size calculations

We used EPI Info software version 6 to determine the sample size required for our study. We assumed that 35% of psoriatic patients and 1% of healthy controls would have increased GLUT1 expression, with 0% makes^5^. Based on these assumptions, we needed at least 25 people in each group.

The study included 74 participants divided into two groups: a healthy control group (G1, n = 30) and a psoriatic group (G2, n = 44). Each participant provided informed written consent. Psoriasis was diagnosed based on skin examination and assessment using the Psoriasis Area and Severity Index (PASI) score. It measures the severity and extent of psoriasis by assessing the redness, thickness, and scaling of psoriasis plaques in four body regions: the head, trunk, arms, and legs. Each region is scored on a scale of 0–4, with 0 indicating no psoriasis and 4 indicating severe psoriasis. The PASI score is then calculated by multiplying the score for each region by the area of the body region affected. The total PASI score can range from 0 to 72, with higher scores indicating more severe psoriasis^17^. Severity was classified as mild, moderate, severe, and very severe when 0 ≤ PASI ≤ 5, 5 < PASI ≤ 12, 12 < PASI ≤ 20, and PASI > 20, respectively^17,18^.

Participants were excluded if they had diabetes mellitus or any other dermatological or systemic disease that affects GLUT1 mRNA expression, such as cancer or diabetic nephropathy^19^. Patients taking systemic treatment were also excluded. All participants underwent a complete medical history and physical examination, including blood pressure measurements and body mass index (BMI) calculation (kg/m^2^)^20^.

### Laboratory work

Under aseptic conditions about 10 ml of venous blood was collected and divided into three tubes; 2 ml was collected as whole blood on fluoride-containing tubes for glycated hemoglobin percentage (HbA1c%) and random blood glucose (RBG) analysis. Four ml was collected on EDTA containing tubes as whole blood and stored at – 80 °C. The other 4 ml was collected in a plain tube, left to clot, centrifuged at 3000 rpm for 15 min, and the sera were retrieved, aliquoted in Eppendorf tubes, and stored frozen at − 80 °C. Mid-stream freshly voided urine samples were taken from all participants to measure urinary albumin to exclude diabetic nephropathy^21^.

Other laboratory investigations including kidney function tests, and liver function tests lipid profiles were performed, for all patients as routine laboratory work in outpatient clinic lab, Assiut University Hospital.

Serum PEDF levels were measured by human PEDF ELISA kit (Cat# SG-11305, Sinogeneclon, China). Serum IL-6, fetuin-A, and PTX3 levels were measured by human ELISA Kit (abcam, Cambridge Biomedical Campus, Cambridge, CB2 0AX, UK); cat# ab 178013, ab269372, and ab214570, respectively.

### Real-time quantitative polymerase chain reaction analysis (RT-PCR)

The frozen blood was allowed to melt on ice to increase total RNA yield^22^. Then as fast as possible the samples were subjected to total RNA extraction using total RNA mini extraction kit (Spin column) (Applied Biotechnology Institute, Inc, San Luis Obispo, CA United States). The blood samples were lysed using RNA lysis buffer which was followed by addition of chloroform to obtain the aqueous layer. The aqueous layer was added to 70% ethanol then the mixture was transferred to a spin column followed by centrifugation and the flow through was discarded. A washing buffer was added to each spin column with discarding the flow through then elution buffer were added to the center of the column followed by centrifugation of the centrifuge tube containing spin-column. Total RNA concentration and purity were tested using a NanoDrop spectrophotometer (BioTeck, Epoch, USA). RNA was then kept at − 80 °C till being used. Total RNA (300 ng) was used for cDNA synthesis using TOPscript TM Reverse Transcriptase (TOPscriptTM cDNA Synthesis Kit, Enzynomics, Daejeon, Korea). For each cDNA preparation, the total volume was 20 μl using a random hexamer primer. The condition used for cDNA synthesis, by thermal cycler (Applied Biosystem, USA), was at 25 °C for 10 min, 50 °C for 60 min, and the reaction was inactivated at 95 °C for 5 min.

Amplification reactions were performed in a volume of 10 μl with 5 µl of PowerUp SYBR Master Mix (Applied Biosystems, USA), 0.3 µl of each of primer forward and reverse, 3.4 µl of RNAase free water, and 1 µl of cDNA template^23^.

The primers used for *GLUT 1*, forward: 5′ GATTCCCAAGTGTGAGTCGC 3′, *GLUT 1,* reverse: 5′ GACATCATTGCTGGCTGGAG 3′ with accession number NM_006516.4^24^. While housekeeping gene detection primer sets were glyceraldehyde-3-phosphate dehydrogenase (GABDH) forward: 5′ CACCACACTGAATCTCCCCT 3′, and GABDH, reverse: 5′ TGGTTGAGCACAGGGTACTT 3′ with accession number NM_001357943.2^25^. Amplification consisted of an initial denaturation step at 95 °C for 10 min followed by 40 cycles of 15 s at 94 °C, 30 s at 58.5 °C, and 30 s at 72 °C. Performance and optimal annealing temperatures of the PCR primers were first tested with gradient PCR. Quantitative PCR was performed using a CFX Connect Real-time PCR Detection System (Bio-Rad, Hercules, CA, USA). The fold changes of *GLUT 1* mRNA were calculated using the 2^−ΔΔCT^ method^26^.

### In vitro treatment of HSF cell lines with different concentrations of PEDF

The normal HSF (ATCC, USA, Cat # CRL-7449) was obtained from NAWAH-Cell Culture Unit, Cairo, Egypt. Cells were maintained in growth medium consisting of Dulbecco’s Modified Eagle Medium (DMEM), high glucose supplemented with 10% fetal bovine serum (FBS), and 1% penicillin/streptomycin (10,000 U/ml) (all from Gibco,USA) in an incubator controlled at 37 °C, 95% humidity, and 5% CO_2_ in the CO_2_ incubator (Contherm mitre 4000, Contherm Scientific, Hutt city, New Zealand). Human recombinant PEDF (Cat # SRP4988) was obtained from Sigma-Aldrich, Germany. The cell lines were treated with PEDF in different concentrations (20, 50, 100, 250, and 500 ng/ml) for 24, 48, and 72 h. Untreated and treated cell lines were then subjected to total RNA extraction and RT-PCR for *GLUT 1* mRNA expression assessment^27^.

### Statistical analysis

The statistical analysis was performed using SPSS version 26.0 (IBM-SPSS, Chicago, IL, USA). After testing the normality, Independent-Samples T-test and one-way analysis of variance (ANOVA) test were carried out to compare means^28^. Categorical variables were compared by Chi-square test^29^. The correlations between the variables were analyzed using the Pearson correlation coefficient test. Gene expression profile modulation was evaluated by comparing the Ct values by 2^−∆∆Ct^ method^26^. The receiver operating characteristic (ROC) curve was used to detect the ability of the studied parameters to differentiate psoriatic patients from non-psoriatic healthy control^30^. Regression analysis in the entire sample was performed to investigate the independent associations of significant variables with the whole blood *Glut 1* mRNA fold changes as dependent variable. p value ≤ 0.05 was considered significant.

### Ethics approval and consent to participate

The study was conducted according to the guidelines of the Declaration of Helsinki and approved by the Institutional Review Board at the Faculty of Medicine, Assiut University [IRB: 17101042]. A written informed consent was obtained from the parents.

## Results

### Age, gender, BMI, and other laboratory findings of studied groups

Nonsignificant differences between the two groups were found in age and sex. The BMI values were significantly higher in G2 than in G1. Although some significant differences were found in liver function tests and serum albumin levels, the values were still within the normal ranges in both groups (Table 1).

**Table 1:** Mean ± SD of different studied parameters in G1 and G2.

	G1 (healthy, n = 30)	G2 (psoriatic, n = 44)	*p* value
Age (years)	30.8 ± 8.8	32.9 ± 12.6	0.397
Gender			
Male	24 (80%)	31 (70.4%)	0.424*
Female	6 (20%)	13 (29.6%)	
BMI (kg/m^2^)	26.7 ± 4.5	29.1 ± 4.7	0.032
sALT (U/l)	12.5 ± 3.4	25 ± 11	< 0.001
sAST (U/l)	14.7 ± 4.0	21 ± 9.6	< 0.001
s-alb (g/l)	4.3 ± 0.64	4 ± 0.5	0.029
T-Pr (g/l)	7.3 ± 0.63	7.1 ± 0.7	0.068
BUN (mg/dl)	14.2 ± 4.3	15 ± 4	0.224
s-cr (mg/dl)	0.86 ± 0.08	0.81 ± 0.2	0.606
HDL-c (mg/dl)	55.8 ± 2.9	54.6 ± 3.9	0.183
LDL-c (mg/dl)	90.9 ± 19.1	97 ± 14.8	0.123
sTC (mg/dl)	169.5 ± 19.1	176.9 ± 13.6	0.076
sTAG (mg/dl)	114.2 ± 16.2	115.4 ± 27.2	0.961
RBG (mg/dl)	112.8 ± 13	108.1 ± 20.5	0.363
HbA1c%	5.7 ± 0.16	5.6 ± 0.5	0.350
*GLUT 1* mRNA fold changes	0.58 ± 0.49	8.2 ± 3.5	< 0.001
PEDF (ng/ml)	34.5 ± 2.1	14.9 ± 5.6	< 0.001
Serum IL-6 (pg/ml)	15.8 ± 2.8	27.5 ± 8.3	< 0.001
Serum fetuin-A (µg/ml)	75.6 ± 7.4	92.1 ± 16.9	< 0.001
Serum PTX3 (ng/ml)	2.3 ± 0.5	3.6 ± 0.7	< 0.001

The independent-samples T-test was used to compare the means between the two groups. *Chi-square was used to compare the difference in sex distribution between the 2 groups.

*SD* standard deviation, *BMI* body mass index, *sAST* serum aspartate transaminase, *sALT* serum alanine transaminase, *s-alb* serum albumin, *T-Pr* total plasma protein, *BUN* blood urea nitrogen, *s-cr* serum creatinine, *HDL-c* high-density lipoprotein cholesterol, *LDL-c* low-density lipoprotein cholesterol, *sTC* serum total cholesterol, *sTAG* serum triacylglycerol, *RBG* random blood glucose, *HbA1c*% glycated hemoglobin percentage, *GLUT 1 *glucose transporter type 1 gene, *mRNA* messenger ribonucleic acid, *PEDF* serum pigment epithelium-derived factor, *IL-6* interleukin-6, *PTX3* pentraxin-3.

Whole-blood GLUT1 mRNA fold changes and serum levels of IL-6, fetuin-A, and PTX3 were significantly higher, while serum PEDF levels were significantly lower, in psoriatic patients than in healthy individuals (Table 1).

### Disease history and PASI score in the psoriatic patients (G2)

The duration of psoriasis in G2 was 6.9 ± 4.9 years. The PASI score values were 16.9 ± 13.5. The G2 participants were categorized according to the PASI score categories into mild [n = 5 (11.3%)], moderate [n = 20 (45.5%)], severe [n = 8 (18.2%)], and very severe [n = 11 (25%)]. Fitzpatrick skin phototypes reported in the current work were type II [n = 2 (4.5%)], III [n = 9 (20.5%)], and IV [n = 33 (75%)].

Of the patients, 37 (84.1%) received topical treatment, and 14 (31.8%) received phototherapy. A family history of psoriasis and atopy was reported in 6 (13.6%) and 10 (22.7%) patients, respectively. None of the patients had psoriatic arthritis.

### Comparison of different studied parameters by the PASI score categories in psoriatic patients (G2)

Whole-blood *GLUT1* mRNA fold changes and serum levels of IL-6, fetuin-A, and PTX3 were significantly higher in the very severe group than in the severe, moderate, and mild groups. Serum PEDF levels were significantly lower in the very severe group than in the severe, moderate, and mild groups (Table 2).

**Table 2:** Comparison of mean ± SD of whole blood *GLUT 1* mRNA fold changes, serum levels of IL-6, fetuin-A, PTX3, and PEDF by the PASI score categories in psoriatic patients (G2).

	PASI score categories				*p* value
	Mild n = 5	Moderate n = 20	Severe n = 8	Very severe n = 11	
*GLUT 1* mRNA fold changes	4.7 ± 0.9	6.6 ± 2.3	9.5 ± 2.2	11.7 ± 3.4	< 0.001
PEDF (ng/ml)	23.7 ± 7.1	16.1 ± 3.8	12.6 ± 2.6	9.9 ± 2.9	< 0.001
Serum IL-6 (pg/ml)	18.9 ± 5.1	24.9 ± 8.9	29.2 ± 1.6	34.7 ± 4.9	< 0.001
Serum fetuin-A (µg/ml)	80.8 ± 15.2	86.6 ± 16.8	91.4 ± 5.1	107.7 ± 4.9	0.001
Serum PTX3 (ng/ml)	2.6 ± 0.5	3.5 ± 0.7	3.7 ± 0.4	4.3 ± 0.4	< 0.001

One-way analysis of variance (ANOVA) test was used to compare the mean between groups. *SD* standard deviation, *PASI* psoriasis area and severity index, *GLUT 1 *glucose transporter type 1 gene, *mRNA* messenger ribonucleic acid, *PEDF* serum pigment epithelium-derived factor, *IL-6* interleukin-6, *PTX3* pentraxin-3.

### Corrections

In the whole study sample, whole-blood *GLUT1* mRNA fold changes were negatively correlated with serum PEDF levels and positively correlated with serum levels of IL-6, fetuin-A, and PTX3. Additionally, serum PEDF levels were negatively correlated with serum levels of IL-6, fetuin-A, and PTX3. Furthermore, there were positive correlations among serum levels of IL-6, fetuin-A, and PTX3 (Table 3).

**Table 3:** correlations among whole blood *GLUT 1* mRNA fold changes, serum levels of PEDF, IL-6, fetuin-A, and PTX3 in the whole study sample.

		Whole blood Glut 1 mRNA fold changes	Serum PEDF (ng/ml)	Serum IL-6 (pg/ml)	Serum fetuin-A (µg/ml)	Serum PTX3 (ng/ml)
Whole blood Glut 1 mRNA fold changes	*r*	1	− 0.828**	0.619**	0.513**	0.699**
	*p*		< 0.001	< 0.001	< 0.001	< 0.001
Serum PEDF (ng/ml)	*r*	− 0.828**	1	− 0.614**	− 0.446**	− 0.654**
	*p*	< 0.001		< 0.001	< 0.001	< 0.001
Serum IL-6 (pg/ml)	*r*	0.619**	− 0.614**	1	0.750**	0.827**
	*p*	< 0.001	< 0.001		< 0.001	< 0.001
Serum fetuin-A (µg/ml)	*r*	0.513**	− 0.446**	0.750**	1	0.721**
	*p*	< 0.001	< 0.001	< 0.001		< 0.001
Serum PTX3 (ng/ml)	*r*	0.699**	− 0.654**	0.827**	0.721**	1
	*p*	< 0.001	< 0.001	< 0.001	< 0.001	

*R *Pearson correlation, *p *p value, *GLUT 1 *glucose transporter type 1 gene, *mRNA* messenger ribonucleic acid, *PEDF* serum pigment epithelium-derived factor, *IL-6* interleukin-6, *PTX3* pentraxin-3.

**Correlation is significant at the 0.01 level (2-tailed).

**Figure 1:**
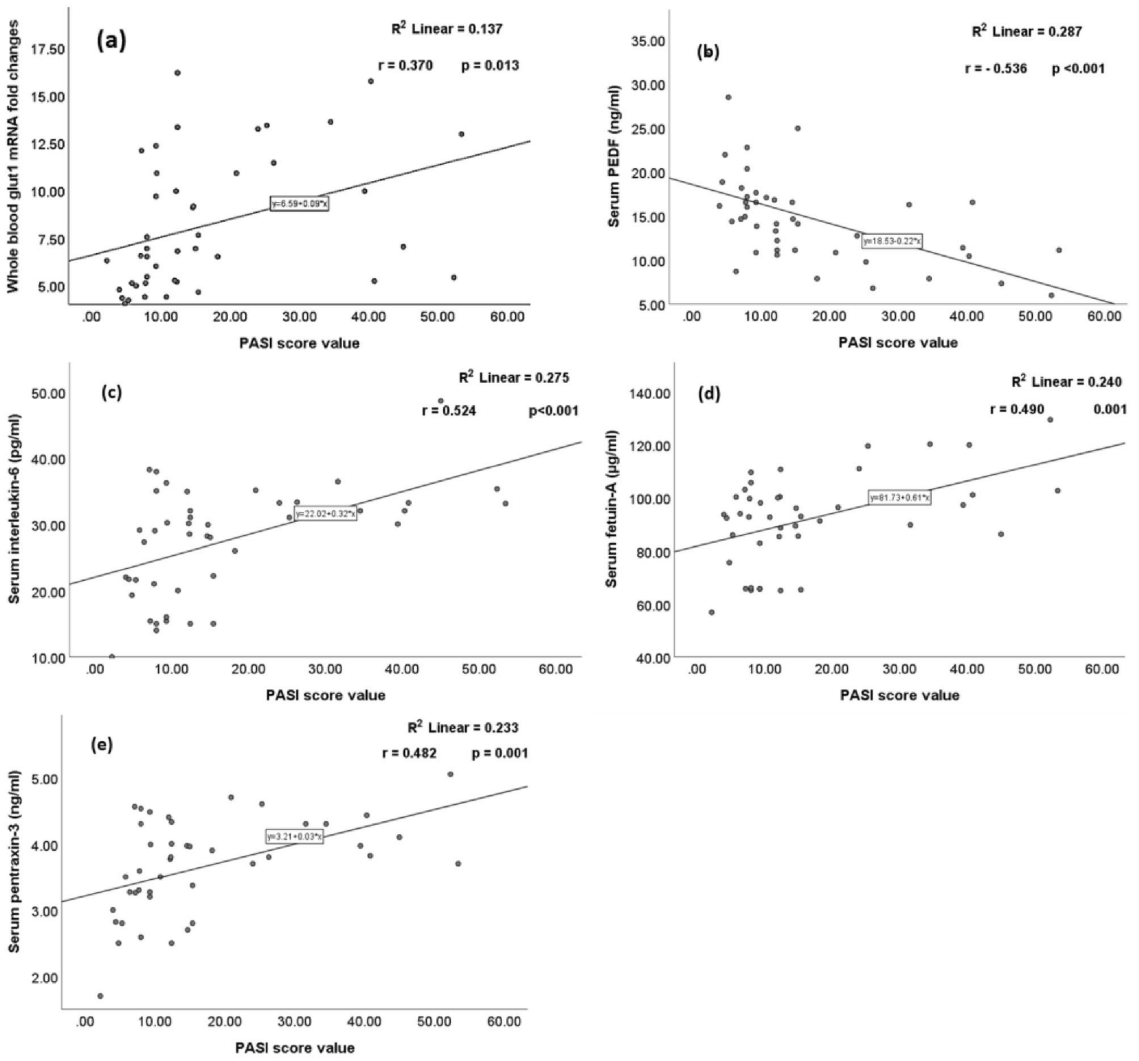
Simple scatters of (**a**) whole blood *glut1* mRNA fold changes, (**b**) serum pigment epithelium-derived factor (PEDF) levels, (**c**) serum interleukin-6 levels, (**d**) serum fetuin-A levels, and (**d**) serum pentraxin-3 levels by PASI score value.

Within the psoriatic group (G2), the Psoriasis Area and Severity Index (PASI) scores correlated positively with whole-blood GLUT1 mRNA fold changes and serum levels of IL-6, fetuin-A, and PTX3, and negatively with serum PEDF levels (Fig. 1).

### Regression analysis

The effect strengths of different studied parameters on the *Glut 1* mRNA fold changes are displayed in Table 4. A significant impact of serum PEDF levels was found.

**Table 4:** Multiple linear regression analysis in the entire sample.

Model		Coefficients^a^					
		Unstandardized coefficients		Standardized coefficients	*p*	95.0% confidence interval for B	
		B	Standard error	Beta		Lower bound	Upper bound
1	(Constant)	12.668	3.155		0.000	6.374	18.962
	Serum PEDF (ng/ml)	− 0.343	0.050	− 0.797	0.000	− 0.443	− 0.243
	Serum IL-6 (pg/ml)	− 0.067	0.071	− 0.126	0.350	− 0.208	0.075
	Serum fetuin-A (µg/ml)	− 0.017	0.030	− 0.061	0.561	− 0.077	0.042
	Serum TX3 (ng/ml)	1.047	0.665	0.211	0.120	− 0.280	2.374

*p *p value, *GLUT 1 *glucose transporter type 1 gene, *mRNA* messenger ribonucleic acid, *PEDF* serum pigment epithelium-derived factor, *IL-6* interleukin-6, *PTX3* pentraxin-3.

^a^Dependent Variable: whole blood *Gut 1* mRNA fold changes.

### ROC curve analysis for the studied parameters

**Figure 2:**
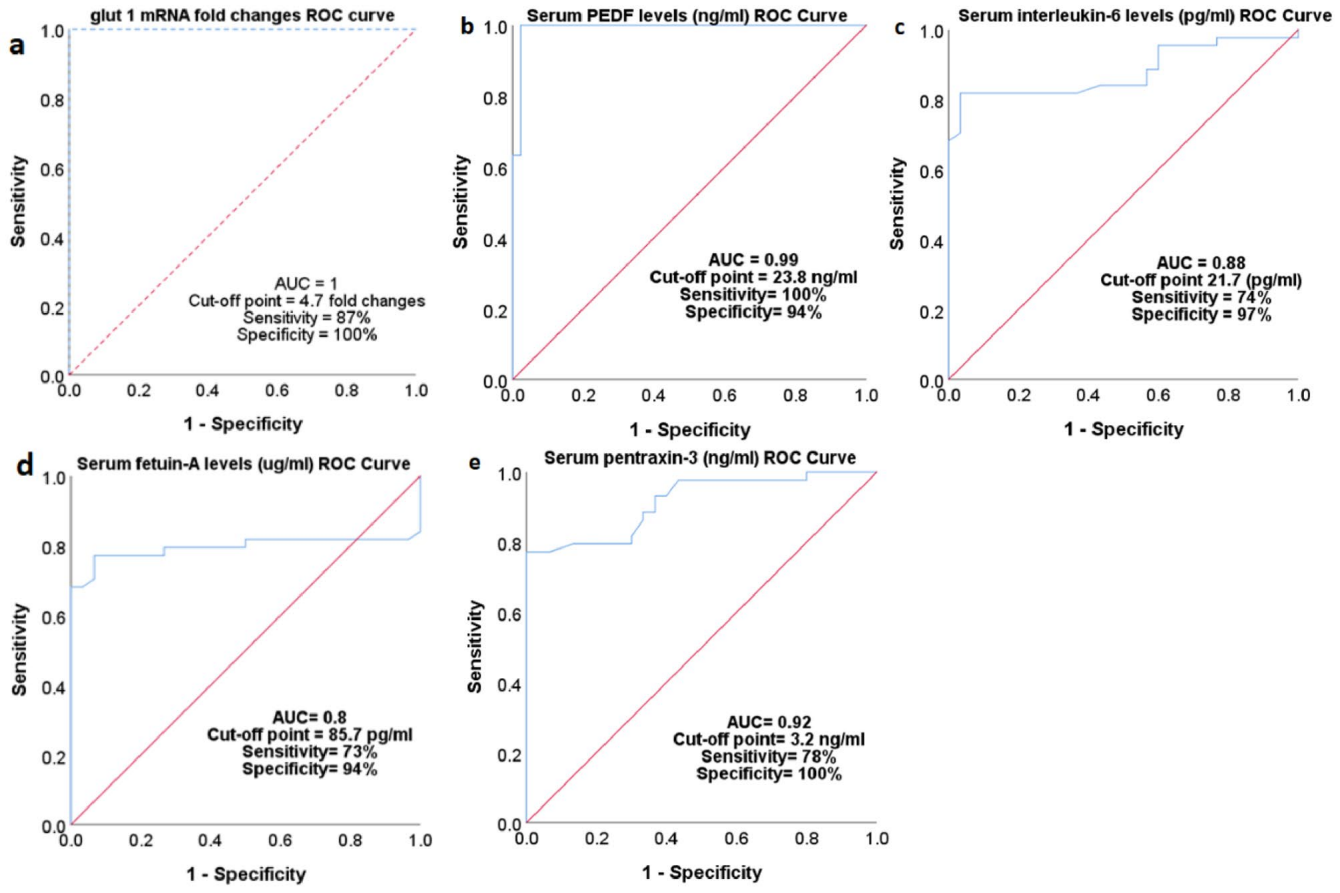
Receiver operating characteristic (ROC) curve for the capability of (**a**) whole blood *GLUT 1* mRNA fold changes (**b**) serum pigment epithelium-derived factor (PEDF) levels, (**c**) serum interleukin-6 levels, (**d**) serum fetuin-A levels, and (**e**) serum pentraxin-3 levels to discriminate psoriatic patients from normal subjects. *AUC* area under the curve.

The AUCs, cut-off points, sensitivities, and specificities of different study parameters for distinguishing cases of psoriasis from healthy individuals are shown in Fig. 2.

### Effect of HSF treatment with PEDF on ***GLUT 1*** mRNA expressions:

**Figure 3:**
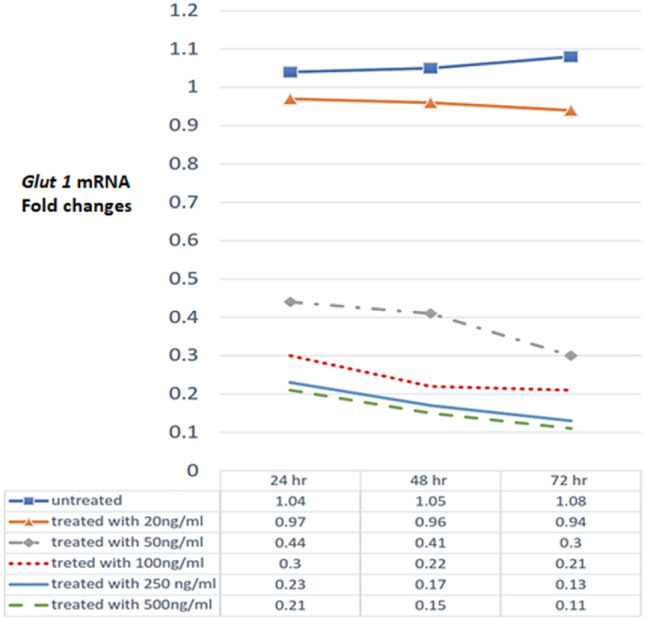
*GLUT 1* mRNA fold changes in in pigment epithelium-derived factor (PEDF)-untreated and treated human skin fibroblast (HSF) cell lines.

Using RT-PCR, *GLUT1* mRNA fold changes were measured in HSF cell lines after treatment with different concentrations of PEDF. In our study, the treated cells showed both dose- and time-dependent decreases in GLUT1 mRNA fold changes (Fig. 3).

## Discussion

To our knowledge, this is the first study to report higher levels of whole-blood Glut1 mRNA (non-invasively) in psoriatic patients. Additionally, the negative impact and reverse correlations between whole-blood Glut1 mRNA and serum PEDF levels, along with the dose- and time-dependent decreases in GLUT1 mRNA fold changes upon treatment of human fibroblast cell lines with PEDF, all suggest a possible therapeutic role for PEDF in psoriatic cases.

Glucose transporters (GLUTs) are transmembrane proteins with twelve membrane-spanning regions that facilitate glucose transport across the cell membrane^31^. Of these GLUTs, glucose transporter type 1 (GLUT1) is an insulin-independent glucose transporter that is present mainly in red blood cells, lymphocytes, brain, and the blood–brain barrier^31,32^. *GLUT 1* is overexpressed in many cancers, allowing the cells to get more glucose for their energy production, growth, and proliferation^33^. Similar findings were reported in psoriatic keratinocytes^7^. Inhibition of GLUT1-mediated glucose uptake might control keratinocyte proliferation^6^. These findings are consistent with the results of the current study.

Also, our results are consistent with the findings of many previous studies^5–8,34^. GLUT1 concentration and gene expression were elevated in skin biopsies from psoriatic lesions compared to non-lesioned skin and normal control skin^5^. In one study, all skin biopsies from psoriatic patients expressed GLUT1 to varying degrees immunohistochemically^34^. Abdou et al. reported that GLUT1 was not expressed in normal control skin but was expressed in 76.6% of uninvolved and 86.7% of involved psoriatic skin^8^. Hodeib et al. reported that GLUT1 mRNA expression correlated positively with PASI score in skin biopsies^5^. Psoriatic lesions with pronounced acanthosis were more likely to express *GLUT1* than lesions with mild to moderate acanthosis^8^. These results are consistent with the findings of the current study, but this study differs in that it used whole blood samples.

On the other hand, the significant positive correlation between duration of psoriasis and GLUT1 mRNA expression found in the current study contradicts the results of Hodeib et al., who reported no correlation between duration of psoriasis and *GLUT1* expression immunohistochemically^5^.

The negative correlation between GLUT1 mRNA fold changes and serum PEDF levels in the current study was consistent with the findings of Calado et al.^35^. Additionally, we reported a positive correlation between *GLUT1* mRNA fold changes and IL-6, fetuin-A, and PTX3 levels, which may indicate the multifactorial nature of psoriasis^36^.

Pigment epithelium-derived factor (PEDF) is a 50-kDa antiangiogenic protein, initially identified for its ability to induce differentiation in retinoblastoma cells^37^. Additionally, PEDF promotes a differentiated, non-proliferative state in various cell types^37^. It also serves as an antioxidant, protecting neurons from light-induced damage and glutamate excitotoxicity^37^. Normal human skin fibroblasts (HSF) express PEDF^38,39^. Consistent with the findings of the current study, PEDF levels were observed to be lower in psoriatic skin biopsies compared to normal skin^40^. The topical application of low molecular weight PEDF peptides in a mouse model of psoriatic disease led to a reduction in the thickness and angiogenesis of skin lesions^10^. In contrast to these findings, Nakajima et al., and Tekely et al. reported higher PEDF levels in the sera of psoriatic patients compared to controls, with no significant correlations between serum PEDF levels and the PASI score^41,42^.

In line with our findings, many previous studies have found high IL-6 levels and gene expression in the blood and skin lesions of psoriatic patients. These levels were associated with the severity of psoriasis and its complications^13,43–45^.

Fetuin-A is a glycoprotein produced by the liver that plays several important roles in the body, including regulating bone metabolism, inhibiting vascular calcification, protecting against inflammation, and regulating insulin activity^46^. Consistent with our results, serum fetuin-A levels were higher in psoriatic patients than healthy individuals and suggested to play a pivotal role in the pathogenesis of psoriasis and its accompanying inflammation and cardiometabolic complications^14,46,47^. On the other hand, some studies have reported lower levels of fetuin-A in psoriatic patients compared to healthy individuals^48^.

The PTX3 is an anti-inflammatory and anti-infection protein that activates immune cells. It has been linked to several diseases, including inflammatory diseases, cardiovascular disease, cancer^49^. PTX3 levels are elevated in psoriatic patients and have been reported as an indicator of psoriasis severity, as well as a useful marker for cardiovascular, metabolic, and joint involvement in these cases^15,50–52^.

The ROC curve analysis in the current study showed high diagnostic accuracies to distinguish psoriatic patients from non-psoriatic healthy people for the whole blood *Glut 1* mRNA fold changes, and serum PEDF and PTX3 levels. The accuracy for the serum IL-6 and fetuin-A levels was only moderate.

The main limitation of the current study is its small sample size.

In conclusion, whole blood *GLUT1* mRNA is a noninvasive parameter that is significantly increased in psoriatic patients. Its expression correlates with the severity of psoriasis, as well as with serum levels of IL-6, fetuin-A, and PTX3. Conversely, serum PEDF levels negatively correlate with whole blood *GLUT1* mRNA expression. In vitro treatment of human fibroblast cell lines with PEDF significantly decreases *GLUT1* mRNA expression in a dose- and time-dependent manner. Further research is necessary to investigate the potential of PEDF as a treatment for psoriasis.

## Data Availability

The data will be shared on reasonable request to the corresponding author.

## Abbreviations

GLUT 1: Glucose transporter 1
PEDF: Pigment epithelium-derived factor,
IL-6: Interleukin-6 PTX3: pentraxin-3
HSF: Normal human skin fibroblast cell line
Hep-G2: A human hepatoma cell line
RT-PCR: Real time polymerase chain reaction
ELISA: Enzyme linked immunosorbent assay
mRNA: Messenger RNA
T2DM: Type 2 diabetes mellitus
PASI: Psoriasis Area Severity Index
SERPINF1/EPC1: Serpin Family F Member 1
IRB: Institutional Review Board
BMI: Body mass index
EDTA: Ethylenediaminetetraacetic acid
HbA1c%: Glycated hemoglobin percentage
RNA: Ribonucleic acid
cDNA: Complementary deoxyribonucleic acid
NM: Accession number links to the mRNA record in the nucleotide database
*GABDH*: Glyceraldehyde-3-phosphate dehydrogenase- forward
DMEM: Dulbecco’s modified eagle medium
FBS: Fetal bovine serum
SPSS: Statistical package for the social sciences
Ct: Cycle threshold
ROC: Receiver operating characteristic
sAST: Serum aspartate aminotransferase
sALT: Serum alanine aminotransferase
salb: Serum albumin
BUN: Blood urea nitrogen
s-cr: Serum creatinine
HDL-c: High density lipoprotein cholesterol
LDL-c: Low density lipoprotein cholesterol
sTC: Serum total cholesterol
sTAG: Serum triacylglycerol
RBG: Random blood glucose
AUC: Area under the curve
